# Intervention of Grape Seed Proanthocyanidin Extract on the Subchronic Immune Injury in Mice Induced by Aflatoxin B1

**DOI:** 10.3390/ijms17040516

**Published:** 2016-04-08

**Authors:** Miao Long, Yi Zhang, Peng Li, Shu-Hua Yang, Wen-Kui Zhang, Jian-Xin Han, Yuan Wang, Jian-Bin He

**Affiliations:** Key Laboratory of Zoonosis of Liaoning Province, College of Animal Science & Veterinary Medicine, Shenyang Agricultural University, Shenyang 110866, China; longjlau@126.com (M.L.); sihuo12345@sohu.com (Y.Z.); lipeng79625@163.com (P.L.); yangshuhua0001@126.com (S.-H.Y.); zhangwenk@126.com (W.-K.Z.); myworld_a4@126.com (J.-X.H.); cywfu2008@163.com (Y.W.)

**Keywords:** grape seed proanthocyanidin extract, AFB1, immune injury, alleviate, mice

## Abstract

The aim was to investigate the prevention of grape seed proanthocyanidin extract (GSPE) on the subchronic immune injury induced by aflatoxin B1 (AFB1) and the possible ameliorating effect of GSPE in mice. The subchronic AFB1-induced immune injury mice model was set up with the continuous administration of 100 μg/kg body weight (BW) AFB1 for six weeks by intragastric administration. Then, intervention with different doses (50 and 100 mg/kg BW) of GSPE was conducted on mice to analyze the changes of body weight, immune organ index, antioxidant capability of spleen, serum immunoglobulin content, and the expression levels of inflammatory cytokines. The prevention of GSPE on the immune injury induced by AFB1 was studied. The GSPE could relieve the AFB1-induced reduction of body weight gain and the atrophy of the immune organ. The malondialdehyde (MDA) level of the spleen in the AFB1 model group significantly increased, but levels of catalase (CAT), glutathione (GSH), glutathione peroxidase (GSH-P_X_), and superoxide dismutase (SOD) significantly decreased. The GSPE could significantly inhibit the oxidative stress injury of the spleen induced by AFB1. AFB1 exposure could not significantly change the contents of IgA, IgG, or IgM. AFB1 significantly improved the expression of interleukin 1β (IL-1β), IL-6, tumor necrosis factor α (TNF-α), and interferon γ (IFN-γ). Additionally, GSPE could decrease the expression of these four proinflammatory factors to different degrees and inhibit the inflammatory reaction of mice. The results suggest that GSPE alleviates AFB1-induced oxidative stress and significantly improves the immune injury of mice induced by AFB1.

## 1. Introduction

Aflatoxins are a group of secondary metabolites produced by strains of some *Aspergillums* species, mainly *A. flavus* and *A. parasiticus*. Among the aflatoxins, aflatoxin B1 (AFB1) is the most biologically active form, and it causes poor performance, liver lesions, immunosuppression, alters blood profiles, and gut morphology, and partially damages internal organ in many animals [1,2,3]. Although *in vivo* effects of AFB1 toxicity vary considerably with the animal species, extent and duration of exposure, age, and nutritional status [4], AFB1 is harmful to animal and human health, mainly due to effects such as increasing free radical production, leading to oxidative damage and lipid peroxidation, which might ultimately lead to cell damage and death [5,6]. As oxidative stress plays a key role in the toxicity mechanism of AFB1; therefore, some antioxidants might be useful in preventing or attenuating the detrimental effects of chronic AFB1 toxicity in animals [7,8].

Grape seed proanthocyanindin extract (GSPE) is derived from grape seeds. They have demonstrated a marked spectrum of biological, pharmacological, therapeutic, and chemoprotective properties against oxygen free radicals and oxidative stress, and they also have shown the ability to mediate anti-inflammatory [9,10]. Many data have shown that the ability of GSPE to improve antioxidant defenses for protecting the main organ function, such as preventing liver injury in the carbon tetrachloride- induced and ischemia/reperfusion-induced [11,12], alleviating Arsenic-induced oxidative reproductive toxicity [13], and protecting the renal function from Cisplatin-induced nephrotoxicity [14]. Recent studies have also shown that GSPE has anti-inflammatory and immunodulatory properties [15,16,17,18,19].

However, it has not been clear whether GSPE could reverse the inflammatory status induced by AFB1. In this study, the effects of AFB1 on oxidative status, immunity, and the expression of inflammation-related genes of spleens in mice were investigated, and whether the treatment with GSPE was able to counteract its negative effects was also studied.

## 2. Results

### 2.1. Effects on Body Weight and Organ Index

**Figure 1:**
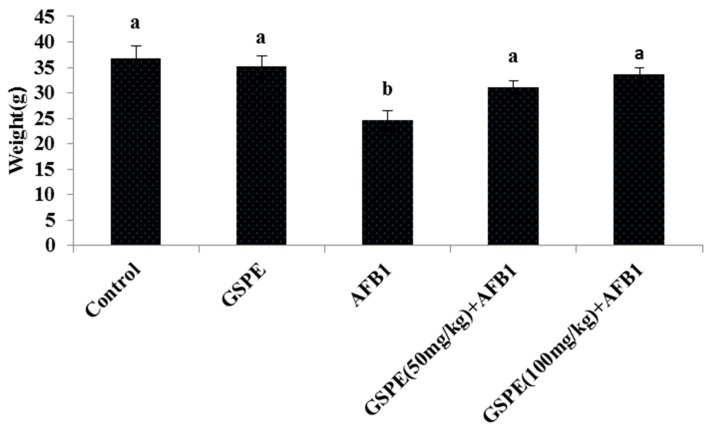
Effect of aflatoxin B1 (AFB1), grape seed proanthocyanidin extract (GSPE) and their co-treatment on weight in mice. Values are mean ± SEM of ten mice in each group. a, b Means with different letters are significantly different (*p* < 0.05).

**Figure 2:**
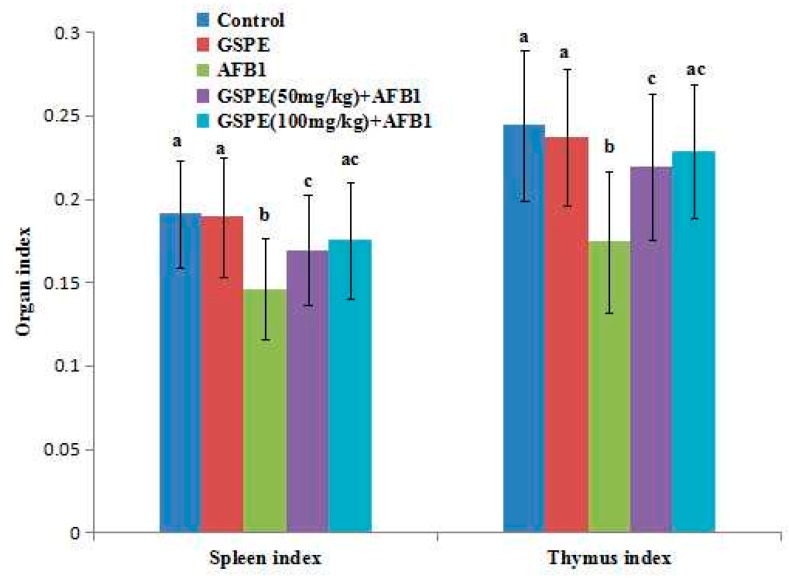
Effect of AFB1, GSPE, and their co-treatment on spleen index and thymus index in mice. Values are mean ± SEM of ten mice in each group. a, b, c Means with different letters are significantly different (*p* < 0.05).

From Figure 1, it can be seen that the body weight in low doses of the AFB1 group was significantly lower than that in the control group at the end of the five-week experiment (*p* < 0.05; Figure 1). However, compared with the AFB1 group, the body weight of mice both in the high-dose GSPE + AFB1 group and the low-dose GSPE + AFB1 group significantly increased (*p* < 0.05; Figure 1). From Figure 2, it can be seen that the spleen index and thymus index significantly decreased in the AFB1 group compared with the control group (*p* < 0.05; Figure 2). However, these indexes increased both in the high and low GSPE + AFB1 group than that in the AFB1 group (*p* < 0.05; Figure 2). These results indicated that AFB1 reduced body weight and caused damage to the immune system. However, the supplementation of GSPE was able to counteract its negative effect on body weight and the immune system.

### 2.2. Effect on Contents of Serum IgA, IgG, and IgM

Compared with the control group, the contents of sera IgA, IgG, and IgM in the AFB1 group did not significantly decrease (Table 1; *p* > 0.05). It indicated that taking 200 μg/kg body weight (BW) AFB1 for five weeks had no significant effect on the humoral immune response. At the same time, GSPE intervention had no significant effect on the content of immunoglobulin.

### 2.3. Effect on Antioxidant of Spleen

Spleen oxidant and antioxidant parameters of mice treatment with GSPE or AFB1 are summarized in Table 2. The administration of AFB1 resulted in a significant increase in spleen malondialdehyde (MDA) content when compared with the control group (*p* < 0.05). Treatment with GSPE inhibited the elevating MDA levels upon AFB1 administration, and the levels of MDA were no different in high dose and low dose GSPE groups (*p* > 0.05). Activities of catalase (CAT), glutathione (GSH), glutathione peroxidase (GSH-P_X_), and superoxide dismutase (SOD) all decreased with AFB1 treatment compared with the control group (*p* < 0.05). However, GSPE improved these antioxidant enzyme activities (*p* < 0.05). These results indicate that GSPE significantly improves the antioxidant activities of the spleen and reduces the serious oxidative damage induced by AFB1.

### 2.4. Effect on Serum Inflammatory Cytokines

**Figure 3:**
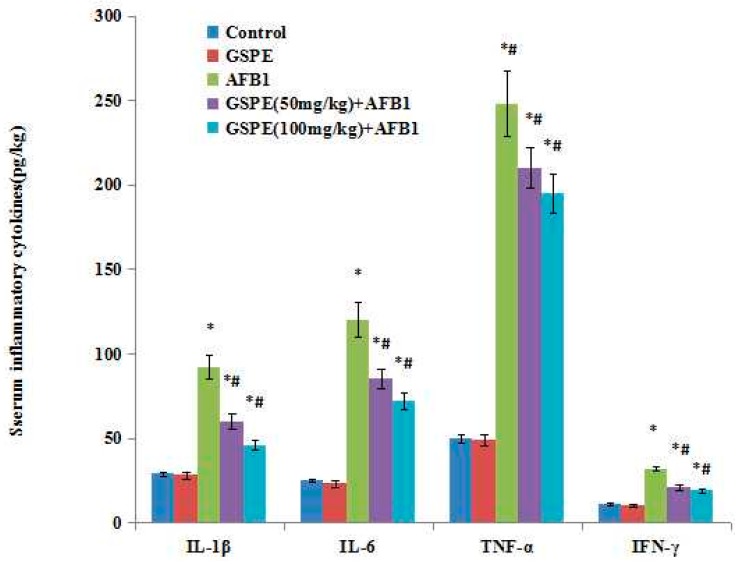
Effect of AFB1, GSPE, and their co-treatment on interleukin 1β (IL-1β), IL-6, tumor necrosis factor α (TNF-α), and interferon γ (IFN-γ) in serum of mice. Values are mean ± SEM of ten mice in each group. ǂ *p* < 0.05 *vs.* control group, # *p* < 0.05 *vs.* AFB1 treated group.

Effect of AFB1 and co-treatment of AFB1 and GSPE on the interleukin 1β (IL-1β), IL-6, tumor necrosis factor α (TNF-α), and interferon γ (IFN-γ) levels in the sera is presented in Figure 3. There was a significant increase in IL-1β, IL-6, TNF-α, and IFN-γ in AFB1-treated mice, as compared to the controls (*p* < 0.05; Figure 3). There was no significant difference between the control and 100-mg/kg GSPE groups (*p* < 0.05), which meant that GSPE had no effect on these inflammatory cytokines. However, co-treatment with AFB1 and GSPE significant decreases the inflammatory cytokines (*p* < 0.05), suggesting that GSPE might be able to downregulate the inflammatory processes occurring in mice serum via AFB1.

### 2.5. Effect on Gene mRNA Expression

**Figure 4:**
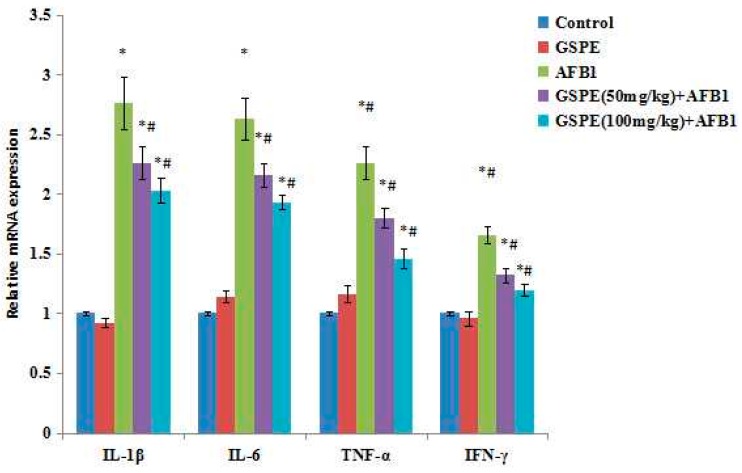
Effect of AFB1, GSPE and their co-treatment on interleukin 1β (IL-1β), IL-6, tumor necrosis factor α (TNF-α), and interferon γ (IFN-γ) relative mRNA expression in spleen of mice. Values are mean ± SEM of ten mice in each group. ǂ *p* < 0.05 *vs.* control group, # *p* < 0.05 *vs.* AFB1 treated group.

The relative mRNA expression of inflammation-related genes was measured via RT-PCR in the spleen samples collected at the end of the experiment (Figure 4). In the administration of the AFB1 in mice, the relative mRNA expressions of IL-1β, IL-6, TNF-α, and IFN-γ were upregulated (*p* < 0.05; Figure 4), up to 2.76 times, 2.63 times, 2.26 times, and 1.65 times, compared to the control group, respectively. However, increased relative expressions of IL-1β, IL-6, TNF-α and IFN-γ genes due to AFB1 were prevented (*p* < 0.05) by the addition of GSPE to the AFB1 (Figure 4). Compared with the AFB1 group, the IL-1β expression amounts were reduced by 18.12% and 26.45%, the IL-6 expression amounts were reduced by 17.87% and 26.62%, the TNF-α expression amounts were reduced by20.35% and 35.40%, and the IFN-γ expression amounts were reduced by 20.00% and 27.88% in the GSPE (50 mg/kg) + AFB1 and GSPE (100 mg/kg) + AFB1 groups, respectively.

## 3. Discussion

At low exposure levels, AFB1 reduced the growth rate, feed efficiency, and the immune response of young animals [20,21]. In this study, five weeks after mice were administrated with AFB1, it was shown that the mice spleen index, thymus index, and body weight in AFB1 significantly decreased, compared with the control group (*p* < 0.05). Overall, AFB1 reduced the growth of the mice, caused tymus of fabricius atrophy, and induced swelling of the spleen, which were consistent with the results reported by Jha *et al.* (2013) [22] and Abbès *et al.* (2016) [23]. We also assessed the effects of GSPE on body weight and immune organ indexes in mice administrated with AFB1. It was shown that, in the group administrated with AFB1 + GSPE, the spleen and thymus indexes, as well as body weights, significantly increased when compared with the AFB1 group (*p* < 0.05). Some researchers reported that AFB1 could induce the intestinal absorbing barrier by reducing the activity of pancreatolipase, amylase, and trypsin and change the energy metabolism of the cell by disturbing the gluconeogenesis, tricarboxylic acid cycle, and fatty acid synthesis, which caused the slow growth of body weight [24,25]. These reports may be the main reason why AFB1 reduced the growth of the mice. Although our results also showed that AFB1 caused a slow increase in the body weight in mice, the body weight that improved with the treatment with the GSPE should be further studied.

AFB1 can induce the production of reactive oxygen species (ROS), which attack the cell membrane lipids and can change the cell membrane fluidity and permeability, resulting in oxidative damage [26,27]. The degrees of the cell damage and lipid peroxidation can be measured by determining the content of MDA, since MDA are the main products of polyunsaturated lipid peroxidation [28]. The GSH, SOD, CAT, and GSH-P_X_ are important components of the endogenous antioxidant defense system, which plays an important role in free radicals scavenging and maintain intracellular redox balance. The intake of AFB1 can cause the oxidative stress by decreasing these antioxidant levels [29]. Our results showed that AFB1 increased the concentration of MDA and decreased the activities of GSH, SOD, CAT and GSH-P_X_ in the spleen. However, GSPE effectively removed ROS, inhibited lipid peroxidation and improved the antioxidant level in the spleen. Thus, the antioxidant effect of GSPE plays an important role to protect the spleen injured by AFB1.

Determinations of the concentrations of serum immunoglobulin such as IgA, IgG, and IgM are the most common methods of testing humoral immune responses [30]. It has been found that AFs can affect humoral immunity. Some other reports showed that the AFB1-exposed diets had a significant effect on the IgA, IgG, and IgM, titer serum concentrations of mice [27], pigs [31], and children [32]. However, in our study, the results showed no significant changes in the serum IgG, IgA, and IgM. Similarly, some studies emphasized that AFB1 did not change the humoral immunity [33]. The different results may have been because the effects of AFB1 on humoral immunity depend on the type, the dose, the duration of exposure, and the susceptibility of each species (e.g., pigs, rats, chicken).

The serum and some tissue production of cytokines were used to further understand immune system status. Under the physiological state, the levels of IL-1β, IL-6, TNF-α, and IFN-γ are lower. However, in pathologic state, these cytokines are secreted and released by tissue cells and have a high level in the serum, resulting in inflammation reaction, and causing tissue damage. The spleen is the body’s largest peripheral immune organ, which plays an important role in inflammation and acquired immune response. Therefore, through the determination, these four cytokines of the spleen can indirectly reflect the immune state of the body. In the current study, the mRNA levels of IL-1β, IL-6, TNF-α, and IFN-γ in spleens were measured by quantitative PCR. The results showed that increased expressions of IL-1β, IL-6, TNF-α, and IFN-γ mRNA were observed in mice exposed to AFB1. The data indicated that a low dose of AFB1 resulted in the inflammation response in spleens, and the inflammatory status was observed in mice spleens. Our current study also showed that GSPE inhibited the production and the expression of the IL-1β, IL-6, TNF-α, and IFN-γ genes of spleens in AFB1-induced mice. In accordance with these observations obtained herein, Lee *et al.* [34], Subarnas *et al.* [35], and Ahmad *et al*. [36] have also demonstrated various anti-inflammatory action of GSPE. All these results also supported the anti-inflammation effects of GSPE, which might be responsible for its effect as a potent antioxidant.

It has been reported that GSPE has been used as a nutritive supplement, a pharmacotherapy, and an antioxidant in foods [37,38]. Indeed, the supplement of GSPE could protect against oxidative stress induced either by certain drugs or under various physiological and pathophysiological conditions [11,12,13,14]. However, there was no report about the prevention of GSPE on the subchronic immune injury induced by AFB1. In the present study, we draw a conclusion that GSPE not only can improve mice weight, the index of immune organs, and the antioxidant ability of spleen, but can also inhibit the expression of inflammatory cytokines. These results might be the main reason that GSPE significantly improves the immune injury of mice induced by AFB1.

## 4. Experimental Section

### 4.1. Animals

Male Kunming mice (18 ± 0.5 g and 4 weeks old) were obtained from the Experimental Animal Center of China Medical University, Shenyang, China. They were bred in a restricted-access room with controlled temperature (22–24 °C), humidity (40%–60%), and 12-h light/dark cycles. The diet and water were given *ad labium*, and all stress factors were reduced to a minimum. Animal experiments were conducted under the principles of good laboratory animal care and with the European Communities Council Directive of 24 November 1986 (86/609/EEC) and were approved by the ethics committee for laboratory animal care and use of Shenyang Agricultural University, China.

### 4.2. Chemicals

AFB1 was purchased from Sigma (St. Louis, MO, USA); GSPE was obtained from Zelang Medical Technology Company (Nanjing, China; purity ≥ 99%). Kits for testing glutathione catalase (CAT), peroxidase (GSH-P_X_), superoxide dismutase (SOD), and malondialdehyde (MDA) activity were purchased from Nanjing Jiancheng Bioengineering Institute (Nanjing, China); the ELISA Kits for testing IgA, IgG, IgM, IL-1β, IL-6, TNF-α, and IFN-γ were purchased from R&D Systems China Co., Ltd. (Shanghai, China); SYBR green RT-PCR kit was acquired from Takara (Otsu, Japan); the RNA sample preservation solution and total animal RNA extraction kits were purchased from Sangon Biotech(Shanghai, China); Revert Aid First Strand cDNA Synthesis Kits were acquired from MBI Fermentas (Burlington, ON, Canada).

### 4.3. Experimental Design

Fifty male Kunming mice were used (18 ± 0.5 g and 4 weeks old). Mice were acclimatized for 1 week before beginning the experiments. These mice were given a standard granulated food and drinking water and were divided into five groups as follows: Group 1: Mice given Oil/water (1:1, *v*:*v*) at 5 mL/kg BW; Group 2: Mice given GSPE at 100 mg/kg b.w. GSPE was at a dose of 100 mg/kg body weight because this was reported to be the most effective dose [39,40]; Group 3: Mice given AFB1 in oil/water (1:1, *v*:*v*) at 100 μg/kg b.w.; Group 4: Mice given 1 h prior to AFB1 administration a dose of GSPE of 50 mg/kg b.w., then the dose of AFB1 100 μg/kg b.w.; Group 5: Mice given 1 h prior to AFB1 administration a dose of GSPE of 100 mg/kg b.w., then the dose of AFB1 100 μg/kg b.w.

During the whole experiment, the body weights of mice were recorded. At the end of the third trial week, the mice fasted for 12 h, and the blood was taken using vacuum vessel via methods of bloodletting. Then, the abdominal cavity was quickly opened, and the spleen and thymus were taken and washed. About 40% of the spleen was homogenized with a Potter (glass-Teflon) in the presence of 10 mM Tris-HCl, pH 7.4 at 4 °C, and centrifuged at 4000 rpm for 30 min at 4 °C. The supernatant was collected for analyzing the antioxidant and the protein concentration was determined. The other 60% of the spleen was preserved in a solution without liquid nitrogen for the subsequent research.

### 4.4. Parameter Analysis

The oxidant levels of the spleen were assessed on the content of MDA, and antioxidant enzyme levels in the spleen were estimated by measuring SOD, CAT, and GSH-P_X_ activities. The analyses were performed using MDA, SOD, CAT, and GSH-P_X_ assay kits.

In the present study, the immunity response status in sera was estimated by measuring the levels of IgA, IgG, IgM, IL-1β, IL-6, TNF-α, and IFN-γ. These indexes were analyzed using the ELISA method. The details of all determination procedures followed the manufacturer’s instructions for the commercial kits (R&D System, Inc., Minneapolis, MN, USA).

### 4.5. Gene Expression Analyses

Total RNA of the testis tissues was extracted using the TRIzol reagent. The total RNA purity was assessed by the quotient of OD at 260/280 nm (1.8–2.0). Reverse transcription was conducted with the TaKaRa PrimeScript RT reagent kit. The mRNA concentrations of spleens for mice IL-1β, IL-2, IL-6, TNF-α, and IFN-γ were quantified by quantitative real-time PCR. β-actin was used as a housekeeping gene to normalize the gene expression data. The primer information for all the genes was selected from the PubMed database (listed in Table 3). Real-time PCR was performed with the SYBR Green PCR Kit and an ABI 7500 real-time PCR system. The measurements of each sample were performed in triplicate. The real-time PCR data were analyzed using the relative gene expression (*i.e.*, 2^−∆∆*C*t^) method. In brief, the data are presented as the fold change in gene expression normalized to the endogenous reference gene (β-actin) and relative to a calibrator.

### 4.6. Statistical Analyses

The variability of results was expressed as the mean ± standard error (X ± SE). The significance of differences between mean values was determined using one-way ANOVA, followed by the least significant difference (LSD) or Student-Newman-Keuls (SNK) *post-hoc* tests for multiple pair-wise comparisons. All statistical tests were calculated using SPSS 13.0 software. Means were considered significantly different at *p* < 0.05.

## Figures and Tables

**Table 1:** Effect of GSPE on immunoglobulin levels in mice induced by AFB1.

Group	IgG (mg/mL)	IgA (mg/mL)	IgM (mg/mL)
Control	15.96 ± 2.31 ^a^	0.54 ± 0.15 ^a^	2.39 ± 0.36 ^a^
GSPE (100 mg/kg)	16.20 ± 2.54 ^a^	0.59 ± 0.22 ^a^	2.36 ± 0.32 ^a^
AFB1 (100 μg/kg)	14.13 ± 2.42 ^a^	0.50 ± 0.28 ^a^	2.30 ± 0.40 ^a^
GSPE (50 mg/kg) + AFB1 100 μg/kg	15.67 ± 2.32 ^a^	0.55 ± 0.29 ^a^	2.35 ± 0.36 ^a^
GSPE (100 mg/kg) + AFB1 100 μg/kg	15.06 ± 2.24 ^a^	0.53 ± 0.24 ^a^	2.32 ± 0.30 ^a^

With each row, means superscript with letter “a” are not significantly different (*p* > 0.05).

**Table 2:** Effect of GSPE on spleen antioxidant parameters in mice induced by AFB1.

Group	MDA nmol/mgprot	CAT U/mgprot	GSH U/mgprot	GSH-P_X_ U/mgprot	SOD U/mgprot
Control	2.82 ± 0.36 ^c^	56.32 ± 3.16 ^c^	279.64 ± 18.14 ^c^	14.60 ± 1.725 ^b^	156.34 ± 23.16 ^b^
GSPE (100 mg/kg)	2.15 ± 0.31 ^c^	75.14 ± 2.96 ^d^	396.74 ± 20.63 ^d^	19.74 ± 1.654 ^c^	214.96 ± 22.34 ^d^
AFB1 (100 μg/kg)	9.87 ± 0.42 ^a^	28.17 ± 2.14 ^a^	196.66 ± 15.43 ^a^	8.842 ± 0.475 ^a^	84.36 ± 20.47 ^a^
GSPE (50 mg/kg) + AFB1 100 μg/kg	5.34 ± 0.38 ^b^	38.75 ± 2.66 ^b^	234.28 ± 18.64 ^b^	12.11 ± 0.362 ^b^	109.14 ± 19.21 ^bc^
GSPE (100 mg/kg) + AFB1 100 μg/kg	4.12 ± 0.29 ^b^	43.23 ± 3.41 ^b^	256.54 ± 19.87 ^b^	13.46 ± 0.330 ^b^	123.45 ± 18.36 ^b^

^a,b,c,d^ Means within the column with different letters are significantly different, *p* < 0.05. MDA: malondialdehyde; CAT: catalase; GSH: glutathione; GSH-P_X_: glutathione peroxidase; SOD: superoxide dismutase.

**Table 3:** Primers for real-time PCR analyses.

Gene	Primer Sequences (5′→3′)	Accession No.	Product Size/bp
β-actin	F: GTGCTATGTTGCTCTAGACTTCG	NM_007393.5	174
	R: ATGCCACAGGATTCCATACC		
IL-1β	F: GAGCACCTTCTTTTCCTTCATCTT	NM_000836	84
	R: TCACACACCAGCAGGTTATCATC		
IL-6	F: GAGGATACCACTCCCAACAGACC	NM_031168	141
	R: AAGTGTATCATCGTTGTTCATACA		
TNF-α	F: ATCCGCGACGTGGAACTG	NM_013693	70
	R: ACCGCCTGGAGTTCTGGAA		
IFN-γ	F: CCATCGGCTGACCTAGAGAA	NM_008337	131
	R: GATGCAGTGTGTAGCGTTCA		
